# Severe paradoxical disease activation following alemtuzumab treatment for multiple sclerosis

**DOI:** 10.1212/NXI.0000000000000799

**Published:** 2020-06-10

**Authors:** Jamie Brannigan, Joanne L. Jones, Sybil R. L. Stacpoole

**Affiliations:** From Jesus College (J.B., S.R.L.S.), Cambridge University, UK; Department of Neurology (J.L.J., S.R.L.S.), Addenbrooke's Hospital, Cambridge University Hospitals NHS Foundation Trust, Cambridge, UK; and Department of Neurology (S.R.L.S.), Peterborough City Hospital, North West Anglia NHS Foundation Trust, Peterborough, UK.

A 39-year-old right-handed agricultural service engineer developed rapidly evolving severe relapsing-remitting multiple sclerosis (MS). MRI showed multiple T2 hyperintensities throughout his neuroaxis (figure, A). Several lesions showed restricted diffusion, and 2 enhanced. He received steroids for each relapse, making a full recovery (Expanded Disability Status Score [EDSS] 0).

**Figure:**
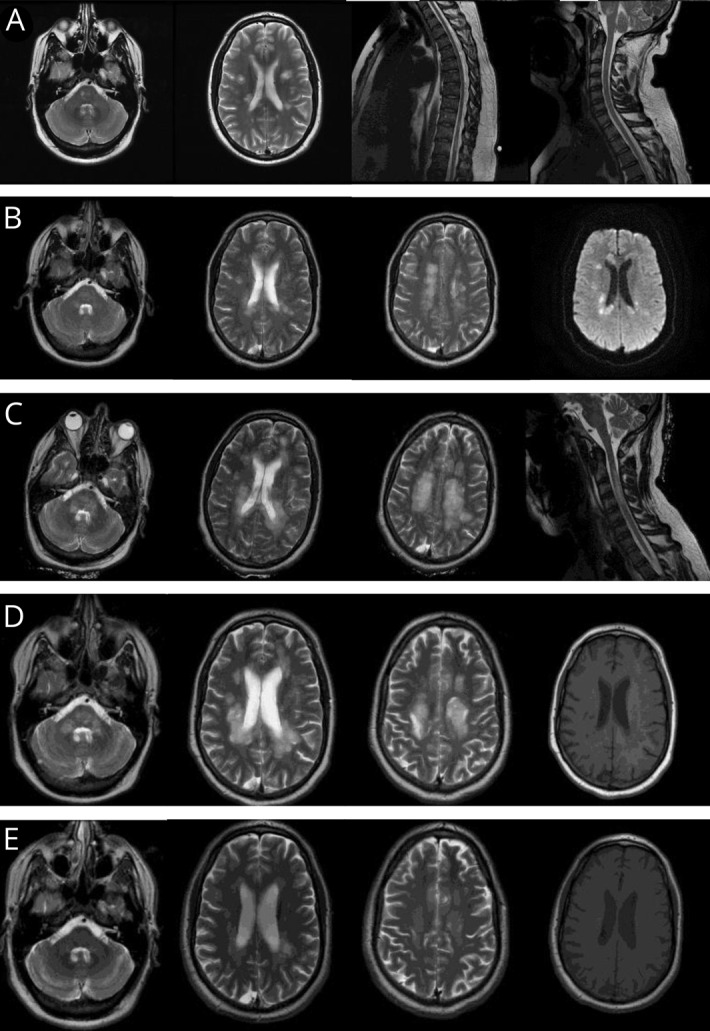
Evolving imaging features at diagnosis, representation, 3 and 5 weeks later, and after 17 months (A) MRI of the brain and spinal cord at the point of diagnosis with rapidly evolving relapsing-remitting MS. His initial demyelinating event involved the brainstem and medial longitudinal fasciculus with several areas of involvement in the cerebral cortex. The second event localized to the lower spinal cord, with no evidence of involvement of the cervical spine. (B) MRI of the brain the day after representation, 9 months after the first cycle of alemtuzumab. T2-weighted imaging showed multiple new areas throughout the brain. Diffusion-weighted imaging confirmed appearances suggestive of acute demyelination with multiple areas of restricted diffusion. (C) MRI of the brain and cervical spine 3 weeks after representation. T2-weighted images show extensive demyelination in the brainstem, cerebral cortex, and cervical spine. (D) MRI of the brain at nadir, 5 weeks after representation. T2-weighted imaging illustrates extensive demyelination with areas of T1 signal change. (E) MRI of the brain 17 months later showing remarkable resolution over time.

There was clinical equipoise between induction therapy with alemtuzumab or natalizumab. He was JC virus positive. He chose treatment with alemtuzumab, partly because of the even chance that after 2 cycles, he might not require further treatment, plus the ease of administration, vs the longer-term risks with natalizumab.

Nine months after the first cycle of alemtuzumab, he represented with an encephalopathic picture and progressive focal neurology. He had a headache for 12 days, diplopia for 8 days, and became confused and unsteady on his feet over 5 days. He was apyrexial, looked unwell, was drowsy, and disorientated. Diplopia was present in all directions, and he had mild left upper motor neurone facial weakness and proximal weakness of his lower limbs. Reflexes were difficult to elicit, with upgoing plantars. He deteriorated markedly over the subsequent week, developing ophthalmoplegia, tetraparesis, inability to communicate, and respiratory compromise requiring intubation (EDSS 9.5).

Serum and CSF markers of infection were all negative, including listeria, but his CSF was active with significantly elevated lymphocytes (200 cells/mm^3^; 90% lymphocytes) and protein (0.85 g/L); glucose was normal. CSF showed unmatched oligoclonal bands. Aquaporin-4 and MOG antibodies were negative.

CSF cytology confirmed reactive lymphocytes (85%). Flow cytometry reported 59% T cells (CD4:CD8 2:1), 35% B cells (kappa:lambda 1.4:1), and 3% NK cells. An additional 13% had a larger morphology and phenotype profile consistent with plasma cells, strongly expressing CD45 and CD38, moderately expressing CD81^+^, and weakly expressing CD19 and light chains.

Repeat brain MRI revealed numerous new white matter lesions. Most showed restricted diffusion, suggestive of acute demyelination (figure, B), but there was no enhancement (postgadolinium images not shown). EEG showed nonspecific generalized slowing, consistent with generalized cerebral dysfunction/encephalopathy.

His presentation was consistent with encephalitis, with an infective or inflammatory cause. Acyclovir, ceftriaxone, and amoxicillin were administered early, followed by 3,000 mg IV methylprednisolone over 3 days and 10 cycles of alternate day plasma exchange.

There was no clinical response, and repeat MRI brain showed progression (figure, C), still without contrast enhancement (not shown). Severe B cell–mediated demyelination was suspected. Rituximab was commenced and continued 6 monthly, alongside 3 cycles of cyclophosphamide. Subsequent gradual improvement resulted in a remarkable functional recovery (EDSS 3.5 two years later with return to full time work) along with significant remyelination and resolution of T1 black holes (figures, D and E).

## Discussion

The first 2 cases of paradoxical disease activation in patients with MS treated with alemtuzumab were reported in 2017.^1^ The authors proposed that a secondary B cell–driven autoimmune disease targeting the CNS and appearing similar to MS could occur after alemtuzumab therapy due to the observation that B-cell numbers recover far more quickly after alemtuzumab than CD4^+^ and CD8^+^ T cells, sometimes overshooting pretreatment levels.^1^ However, the relative kinetics of B- and T-cell recovery have no effect on the risk of non–CNS-directed autoimmunity. Rather, the risk of developing Graves disease and immune thrombocytopenia after alemtuzumab is associated with poor thymic T-cell recovery and exaggerated CD4^+^ T-cell homeostatic proliferation.^2^ CNS-directed alemtuzumab-induced autoimmunity is also unlikely to be due to numerical differences in B- and T-cell counts. Instead, this acute disseminated encephalomyelitis-like illness after alemtuzumab is more likely to result from the complex interplay of multiple reconstituting immune cell subsets.

Eleven of the other 17 reported cases of disease activation after alemtuzumab occurred in those switching therapies directly from fingolimod,^1,3,4^ suggesting confounding of pathologic mechanisms. Following treatment with fingolimod, many lymphocytes remain hidden from the intravascular therapeutic effects of alemtuzumab due to selective lymphoid sequestration. Later egression could initiate rebound activity, which also occurs if fingolimod treatment is stopped without substituting a different therapy.^3^ This disease activity would be due to a failure of alemtuzumab to bind to CD52^+^ lymphocytes, not the repopulation kinetics of B and T cells after treatment.

One question on encountering paradoxical disease activation after alemtuzumab is whether to proceed to the second treatment cycle or switch to rituximab (or an alternative anti-CD20 B-cell therapy such as ocrelizumab). Five patients in the literature received rituximab; 6 including ours. All have responded well. Willis' case series of disease activity 4–5 months post-alemtuzumab in patients switched from fingolimod responded well to the second cycle of alemtuzumab, as might be expected from the mechanisms discussed above.

We hope that the remarkable recovery of our patient, despite extensive brainstem involvement, will provide clinicians with the confidence to treat in such severe situations.
